# Synthetic Alkaloid Treatment Influences the Intestinal Epithelium and Mesenteric Adipose Transcriptome in Holstein Steers

**DOI:** 10.3389/fvets.2020.00615

**Published:** 2020-09-11

**Authors:** Kyle J. McLean, Ransom L. Baldwin, Cong-jun Li, James L. Klotz, J. Lannett Edwards, Kyle R. McLeod

**Affiliations:** ^1^Ruminant Nutrition Laboratory, Department of Animal and Food Sciences, University of Kentucky, Lexington, KY, United States; ^2^Department of Animal Science, University of Tennessee Institute of Agriculture, Knoxville, TN, United States; ^3^Beltsville Agricultural Research Center, Agricultural Research Service, United States Department of Agriculture, Beltsville, MD, United States; ^4^Forage-Animal Production Research Unit, Agricultural Research Service, United States Department of Agriculture, Lexington, KY, United States

**Keywords:** adipose, alkaloids, intestine, ruminants, transcriptome

## Abstract

Holstein steers (*n* = 16) were used to determine if a synthetic alkaloid, bromocriptine, would alter the transcriptome of the small intestine and adjacent mesenteric adipose. On d 0, steers were assigned to one of two treatments: control (CON; saline only) or bromocriptine (BROMO; 0.1 mg/kg BW bromocriptine mesylate injected intramuscularly every 3 d for 30 d). Steers were slaughtered and midpoint sections of jejunal epithelium and associated mesenteric fat were collected for RNA isolation. Transcriptome analysis was completed via RNA-Seq to determine if BROMO differed compared with CON within intestinal epithelium or mesenteric adipose mRNA isolates. Differential expression thresholds were set at a significant *P*-value (*P* < 0.05) and a fold change ≥ 1.5. Only two genes were differentially expressed within the intestinal epithelium but there were 20 differentially expressed genes in the mesenteric adipose tissue (six up regulated and 14 down regulated). Functions related to cell movement, cell development, cell growth and proliferation, cell death, and overall cellular function and maintenance were the top five functional molecular categories influenced by BROMO treatment within the intestinal epithelium. The top molecular categories within mesenteric adipose were antigen presentation, protein synthesis, cell death, cell movement, and cell to cell signaling and interaction. In conclusion, BROMO treatment influenced the intestinal epithelium and mesenteric adipose transcriptome and identified genes and pathways influential to the effects associated with alkaloid exposure which are important to beef production.

## Introduction

Tall fescue (*Lolium arundinaceum*) makes up a sizable portion of the grazing land in the United States and can reduce costs due to increased persistence and drought tolerance. The hardiness of tall fescue is attributed to the presence of an endophyte infection (*Epichloë coenophiala*). This symbiotic relationship between the plant and endophyte increases the sustainability of pastures and increases tall fescues usefulness as a grazing feed source (1). However, tall fescue infected with endophyte decreases animal productivity due to ergot alkaloid ingestion from the endophyte infected plants (2, 3). Ingestion of ergot alkaloids from the plant itself (4, 5) and alkaloids produced by the fungi that infect tall fescue (6) have been implicated in fescue toxicosis. Animals experiencing fescue toxicosis exhibit a variety of symptoms such as increased respiration, impaired immune function, reduced feed intake, and decreased weight gain (7–10). Cattle grazing tall fescue exhibit an unhealthy appearance even after access to fescue has ceased but can experience compensatory gains with increased diet quality (9). Stuedemann and Hoveland (11) proposed that fescue toxicosis can alter lipid metabolism, adipose composition, and increase the occurrence of necrotic fat depots. However, instances of necrotic fat have only been reported in the abdominal fat depots (8) and mainly in older cattle which have grazed endophyte infected fescue over long periods of time.

Ergot alkaloids have been reported to inhibit prolactin secretion in cattle (12, 13). However, ergot alkaloid levels vary greatly in feedstuffs and make consistent exposure difficult in experimental procedures. Bromocriptine is a dopamine receptor agonist that effectively inhibits prolactin secretion (14) similar to ergot alkaloids. Lactating cows fed endophyte-infected fescue or treated with bromocriptine had over 90% of differentially expressed genes (>850 genes) influenced in a similar manner when compared with control cows (15). Doses of bromocriptine have ranged from 15 to 80 mg/animal, and treatment frequencies have ranged from two times a day to three times per week (16–20) but provide a model to decrease prolactin and initiate fescue toxicosis symptoms in cattle with much greater control by not relying on animal intake. Thus, the hypothesis of this study was that ergot alkaloid exposure via bromocriptine treatment would impact the transcriptome of the intestinal epithelium and mesenteric adipose tissue relevant to beef production of growing Holstein steers without impacting feed intake.

## Materials and Methods

All animal procedures were conducted with approval from the Institutional Animal Care and Use Committee at the University of Kentucky (Protocol # 01065A2006).

### Animals

Holstein steers [*n* = 16; initial body weight (BW) 313 ± 16 kg] were used to determine if the synthetic alkaloid, bromocriptine, at levels known to induce fescue toxicosis symptoms (20) would alter the transcriptome of the jejunal epithelium and the adjacent mesenteric adipose. Steers were housed in individual pens (3 × 3 m) within a temperature-controlled research facility and allowed 3 d to adjust to being housed indoors prior to the initiation of the study (d 0). Steers were maintained on a total mixed ration of 61% corn silage, 24% cracked corn, 5% dry distiller's grains with solubles, and 10% soybean meal supplement with 150 mg/head/d rumensin. The overall diet was 49% dry matter, had a net energy for maintenance of 0.367 Mcal/kg, contained 13% crude protein, and was fed to provide 1.5 × net energy for maintenance daily. Maintenance was determined by multiplying empty BW raised to the 0.75 power by 0.77 and increased by 20% to address the increased maintenance energy requirement for dairy animals (21).

On d 0 steers were randomly assigned to one of two treatments: control (CON) or bromocriptine (BROMO). Control animals were intramuscularly injected in the injection triangle of the neck with saline and ethanol (60 and 40%, respectively) at a similar volume to BROMO (~5 mL) every 3 d to coincide with bromocriptine injections. Animals who were assigned to BROMO received bromocriptine mesylate (Santa Cruz Biotechnology; Dallas, TX) at 0.1 mg/kg BW every 3 d beginning on d 0 and ending on d 27 resulting in 10 intramuscular injections. Bromocriptine was reconstituted as working stock in 95% ethanol and diluted with saline so no more than 40% ethanol was given in a single injection. The concentration of BROMO was determined in a previous study where BROMO give at this dosage elicited the same gene expression response in 90% of differentially expressed genes in mammary tissue (*n* = 866) identified comparing the BROMO treatment response with the response observed in cows fed endophyte-infected fescue seed (15). Body weights were taken weekly to ensure feed was provided at 1.5 × maintenance and injection volumes calculated properly. Feed and water intake was individually provided and recorded daily. Orts were weighed daily prior to feeding to calculate daily intake for the previous 24 h. Steers were kept on automatic waterers that were equipped with a water meter to capture water intake. Daily water intake was recorded prior to feeding. Circulating levels of prolactin were used to confirm treatment effect on animal did in fact induce similar effects to fescue toxicosis. Blood samples were taken prior to feeding via jugular venipuncture with 10 mL vacutainer tubes (Becton Dickinson Co.; Franklin Lakes, NJ) upon entry into the housing facility (d −3) and d 0, 7, 14, 21, and 28 to ensure BROMO treated steers had decreased systemic prolactin concentrations. Serum prolactin concentrations were quantified via radioimmunoassay according to procedures previously described (22). The intra and inter assay CVs were 5.35 and 4.37%, respectively, with a sensitivity of 0.05 ng/mL. Prolactin concentrations were analyzed within the mixed procedure of SAS (SAS Institute, Cary, NC) with repeated measures and a compound symmetry covariance structure. The model included treatment, day and the interaction of treatment×day and block as a random covariate. The repeated statement had day as the variable and animal within block as the subject. Least square means were used to separate main effect means with significance set as *P* < 0.05.

On d 30, at 07:00 h the steers were transported 26.7 km from the animal research center beef unit to the meats laboratory at the University of Kentucky. After exsanguination the gastrointestinal tract was removed from the body cavity as quickly as possible (<10 min). The gastrointestinal tract was separated into the forestomach complex (rumen, reticulum, omasum, and abomasum), small intestine, and large intestine. The small intestine was looped over a peg board and a 10 cm section was removed 7 m from the cranial end. Mesenteric adipose adjacent to the 10 cm intestinal section was also removed for further processing. Following separation from the entire tract, 50 mg of adipose tissue submerged in RNAlater (Thermo Fisher Scientific; Waltham, MA) and flash frozen in liquid nitrogen. The small intestinal lumen was exposed, the epithelium was scraped away from the other intestinal tissue layers, and 50 mg of epithelium was submerged in RNAlater (Thermo Fisher Scientific) and flash frozen in liquid nitrogen.

### RNA Isolation and Library Construction

Both jejunum and mesenteric adipose samples were isolated using RNeasy Plus Mini Kit (Qiagen, Valenica, CA). Quality check was performed using Tapestation RNA HS Assay (Agilent Technologies, Santa Clara, CA) and quantified by Qubit RNA HS assay (ThermoFisher Scientific, Waltham, MA). The RNA integrity number for samples was lower and more variable than expected (2.6 ± 0.6 for intestine and 5.0 ± 1.3 for adipose); thus, ribosomal RNA depletion was performed with Ribo-zero Magnetic Gold Kit (Illumina Inc., San Diego, CA) to ensure RNA isolated was of the highest possible quality. Samples were randomly primed and fragmented based on manufacturer's recommendation (NEBNext® Ultra™ RNA Library Prep Kit for Illumina® Illumina Inc., San Diego, CA). The first strand was synthesized with the Protoscript II Reverse Transcriptase for 40 min at 42°C. Synthesis of second strand cDNA was then carried out and products were amplified by PCR to create the cDNA library for each sample. All remaining steps for library construction were done according to the NEBNext® Ultra™ RNA Library Prep Kit for Illumina® (Illumina Inc.). Illumina 8-nt dual-indices were used. Samples were pooled by animal and sequencing was done on a HiSeq with a read length configuration of 150 paired end base pairs with up to 50 million reads per sample.

### Bioinformatic Analysis

Intestinal and adipose library preparation and sequencing, was performed using FASTQC (version v0.11.3). A splice aware aligner, STAR (version 2.5.2b), was used to perform RNA-Seq alignment. The UMD3.1 and UMD3.1.90 from Ensembl were used as genome reference and annotation reference, respectively, during the alignment. Then dupRader and Picard CollectRnaSeqMetrics (version 2.10.5) were used to evaluate duplicates level and overall alignment performance. HT-Seq (version 0.0.6) was used to calculate the per gene expression count. DE-Seq2 was used to estimate differentially expressed genes, all genes are listed in Supplementary Table 1. This list of genes was uploaded into Ingenuity Pathway Analysis (IPA, Qiagen) for further analyses (Supplementary Table 2).

### Ingenuity Pathway Analysis

Differentially expressed genes from RNA-Seq were analyzed via IPA according to previously described methods (23–25). The RNA-Seq data was used by IPA to identify new gene targets, biological mechanisms, pathways, and functions, and candidate biomarkers. Datasets uploaded into the IPA software consisted of all genes identified as significant with a *P* ≤ 0.10 (Supplementary Table 2). From these datasets the most relevant genes were identified as differentially expressed genes (DEG). To qualify as a DEG, genes had to have a significant *P*-value and a positive or negative fold change of >1.5. Canonical pathway analysis identified the most significantly influenced canonical pathways from the IPA library within each tissue. Genes related to each canonical pathway were considered for further analysis. Two measurements are evaluated for canonical pathway analysis (1) the ratio of the number of genes from the data set that map to the pathway divided by the total number of genes that map to the canonical pathway and (2) the Fisher's exact test was used to calculate a *P-*value for the genes in the dataset and the identified canonical pathway. Networks of DEG were then algorithmically generated based on their connectivity. These genes were overlaid onto a global molecular network from the IPA library. Functional analysis identified biological functions associated with DEG that were most significantly influenced by treatment within a tissue. A right-tailed Fisher's exact test was used to calculate a *P-*value for each biological function and/or disease.

## Results

**Figure 1:**
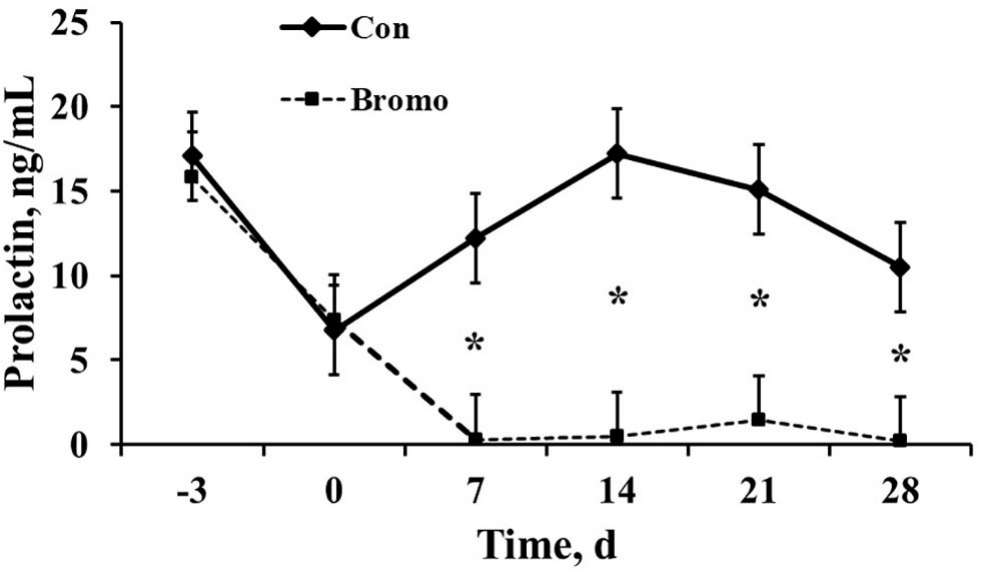
Circulating prolactin concentrations in plasma of control (CON) and bromocriptine (BROMO) treated steers. BROMO prolactin means were different from CON within a given day (*P* < 0.05) and are depicted by an * (d 7, 14, 21, and 28, respectively). Time is in relation to the initiation of BROMO treatments (*d* = 0). The standard error for all data was 2.64.

Circulating prolactin concentrations were similar (*P* > 0.11) between BROMO and Con steers prior to treatment initiation, d −3 and 0 (Figure 1). However, as expected, plasma concentrations were dramatically reduced (*P* = 0.003) in BROMO steers compared with CON steers at all-time points following bromocriptine administration, d 7, 14, 21, and 28.

Threshold for determination of DEG was set at a significant *P-*value (*P* < 0.05) and a fold change of >1.5. Based on selection criteria, RNA-Seq analysis revealed 15 DEG that were down regulated in BROMO compared with CON; one DEG within the intestinal epithelium, fatty acid binding protein 4, and 16 DEG in the mesenteric adipose tissue (Table 1). Analysis identified 7 DEG were identified to be up regulated in BROMO compared with CON; 6 in the mesenteric and UDP-GlcNAc:betaGal Beta-1,3-N-Acetylglucosaminyltransferase in the intestinal epithelium (Table 2).

**Table 1:** Differentially expressed genes^*^ that were down regulated after exposure to Bromocriptine in the intestinal epithelium and mesenteric adipose tissue of Holstein steers.

**Symbol**	**Gene name**	**Fold change**	**Tissue type**	***P-*value**	**Location**	**Type**
FABP4	Fatty Acid Binding Protein 4	1.842	Intestinal	0.020	Cytoplasm	Transporter
CYP4F2	Cytochrome P450 Family 4 Subfamily F Member 2	5.448	Adipose	0.048	Cytoplasm	Enzyme
C8A	Complement C8 Alpha Chain	5.14	Adipose	0.019	Extracellular	Other
ASIC5	Acid Sensing Ion Channel Subunit Family Member 5	5.021	Adipose	0.041	PM^†^	Ion Channel
GLT1D1	Glycosyltransferase 1 Domain Containing 1	4.965	Adipose	0.0278	Extracellular	Enzyme
SERPINB2	Serpin Family B Member 2	4.924	Adipose	0.053	Extracellular	Other
AICDA	Activation Induced Cytidine Deaminase	4.902	Adipose	0.040	Cytoplasm	Enzyme
CDH16	Cadherin 16	4.83	Adipose	0.040	PM	Enzyme
CXCL2	C-X-C Motif Chemokine Ligand 2	4.436	Adipose	0.039	Extracellular	Cytokine
CHGB	Chromogranin B	4.356	Adipose	0.023	Extracellular	Other
SLC26A5	Solute Carrier Family 26 Member 5	4.037	Adipose	0.029	PM	Transporter
HEPACAM2	HEPACAM Family Member 2	4.014	Adipose	0.050	Cytoplasm	Other
NR1H4	Nuclear Receptor Subfamily 1 Group H Member 4	3.862	Adipose	0.019	Nucleus	LDNR^‡^
SLC9A2	Solute Carrier Family 9 Member A2	3.628	Adipose	0.027	PM	Transporter
LEAP2	Liver Enriched Antimicrobial Peptide 2	3.473	Adipose	0.051	Extracellular	Other
DTHD1	Death Domain Containing 1	2.326	Adipose	0.054	Other	Other
CD8A	CD8a Molecule	1.535	Adipose	0.042	PM	Other

* *Differentially expressed genes listed above were limited to those with a P-value <0.05 and >1.5 fold change*.

† *PM, Plasma Membrane*.

‡ *LDNR, ligand-dependent nuclear receptor*.

**Table 2:** Differentially expressed genes^*^ that were up regulated after exposure to Bromocriptine in the intestinal epithelium and mesenteric adipose tissue of Holstein steers.

**Symbol**	**Gene name**	**Fold change**	**Tissue type**	***P-*value**	**Location**	**Type**
B3GNT6	UDP-GlcNAc:betaGal Beta-1,3-N-Acetylglucosaminyltransferase 6	2.853	Intestinal	0.006	Cytoplasm	Enzyme
MOXD1	Monooxygenase DBH Like 1	1.772	Adipose	0.040	Cytoplasm	Enzyme
SORCS2	Sortilin Related VPS10 Domain Containing Receptor 2	1.833	Adipose	0.047	PM^†^	Transporter
SLC11A1	Solute Carrier Family 11 Member 1	1.988	Adipose	0.043	PM	Transporter
LY6G5B	Lymphocyte Antigen 6 Family Member G5B	2.042	Adipose	0.050	Other	Other
DNER	Delta/Notch Like EGF Repeat Containing	3.558	Adipose	0.027	PM	Transmembrane Receptor
PF4	Platelet Factor 4	5.393	Adipose	0.007	Extracellular Space	Cytokine

* *Differentially expressed genes listed above were limited to those with a P-value <0.05 and >1.0 fold change*.

† *PM, Plasma Membrane*.

The canonical pathways influenced by BROMO treatment are listed in Table 3. These pathways were determined to be influenced from the all significant (*P* < 0.05) genes with and without a 1.5 fold change within the datasets (BROMO and CON) via IPA library. In the intestinal epithelium and mesenteric adipose, LXR/RXR activation, granulocyte adhesion, and diapedesis were depressed (*P* < 0.001) in BROMO steers. Atherosclerosis and high mobility group box 1 (HMGB1) signaling and the inflammasome pathway were also depressed (*P* < 0.001) in the intestinal epithelium of BROMO steers compare with CON steers. In the mesenteric adipose, LPS/IL-1 mediated inhibition of RXR function, FXR/RXR activation, antigen presentation, IL-6 signaling, dendritic cell maturation, and serotonin degradation were all depressed (*P* < 0.001) by BROMO treatment (Table 3).

**Table 3:** Canonical pathways influenced by treatment with bromocriptine in the intestinal epithelium and mesenteric adipose in Holstein steers.

**Pathway**	***P*-value**	Ratio^*^	**Genes^†^**
**Intestinal epithelium**
LXR/RXR Activation	<0.0001	0.033	↓CCL2, ↓IL18, ↓LPL, ↓MMP9
Atherosclerosis Signaling	<0.0001	0.031	↓CCL2, ↓IL18, ↓LPL, ↓MMP9
Inflammasome Pathway	0.0001	0.100	↓IL18 and ↑NLRP1
HMGB1^‡^ Signaling	0.0001	0.023	↓CCL2, ↓IL18, ↑PLAT
Granulocyte Adhesion and Diapedesis	0.0003	0.017	↓CCL2, ↓IL18, ↓MMP9
**Mesenteric adipose**
LPS/IL-1 Mediated Inhibition of RXR Function	<0.0001	0.054	↓ABCB11; ↓ALAS1; ↓ALDH1L1, 8A1; ↓FABP7; ↓IL1A; ↓NR1H4; ↓PAPSS2; ↓PLTP; ↓SULT2A1;
			↑IL1RL1; ↑SULT1A1
FXR/RXR Activation	<0.0001	0.071	↓ABCB11; ↓APOA4, B; ↓IL1A; ↓NR1H4; ↓PLTP; ↓SDC1; ↓SULT2A1; ↑SERPINF1
Antigen Presentation Pathway	<0.0001	0.132	↓B2M; ↓PSMB8, 9; ↓TAP1, BP
Granulocyte Adhesion and Diapedesis	<0.0001	0.050	↓CCL8; ↓CXCL2; ↓IL1A; ↓MMP1; ↓SDC1; ↓XCL2; ↑CCL11; ↑IL1RL1; ↑PF4
LXR/RXR Activation	0.0001	0.058	↓APOA4, B; ↓IL1A; ↓NR1H4; ↓PLTP; ↑IL1RL1; ↑SERPINF1
Serotonin Degradation	0.0051	0.052	↓ADH4; ↓ALDH1L1; ↓SULT2A1; ↑SULT1A1
Dendritic Cell Maturation	0.0310	0.026	↓B2M; ↓FCGR3A, B; ↓IL1A; ↑COL1A1; ↑NFKBIA
IL-6 Signaling	0.0284	0.031	↓IL1A; ↑COL1A1; ↑IL1RL1; ↑NFKBIA

* *Ratio: The number of genes differentially expressed within the pathway divided by the total number of genes within the pathway*.

† *Genes that exhibited directional influence by treatment within a pathway*.

‡ *HMGB1: High Mobility Group Box 1. The ↑ arrow is for genes that were up-regulated and the ↓ arrow is for a gene that was down-regulated*.

The top 5 upstream regulators were different between the intestinal epithelium and mesenteric adipose (Tables 4, 5). However, upstream regulation by IL 1A, IL 2, IL 4, IL 6, IL 18, IGF 1, growth hormone (GH), extracellular signal–regulated kinases 1/2 (ERK1/2), and toll-like receptor 4 (TLR 4) were found in the top 30 upstream regulators for both tissue types. In the intestinal epithelium, regulator of G protein signaling (RGS2), MAPK 9, prostaglandin-endoperoxide synthase 2 (PTGS2), proteinase 3 (PRTN3), and histidine rich glycoprotein (HRG) were found to be the top 5 upstream regulators (Table 4). In the mesenteric adipose, TNF, interferon α receptor (IFNαR), IL 1B, NF-κβ, and IFNγ were found to be the top five upstream regulators (Table 5). Also, prolactin, which is depressed by BROMO treatment (Figure 1), was identified as an upstream regulation of the transcriptome in the mesenteric adipose of BROMO steers (Table 5).

**Table 4:** Upstream regulators in the intestinal epithelium and how they influence gene expression.

**Genes^†^**	Upstream Regulators^*^															
	**Top 5**					**IL1A**	**IL2**	**IL4**	**IL6**	**IL18**	**ERK 1/2**	**IGF 1**	**GH**	**IFNαR**	**TLR4**	**IFNγ**
	**RGS2**	**MAPK 9**	**PTGS 2**	**PRTN 3**	**HRG**											
CCL2		↓	↓	↓	↓	↓	↓	↓	↓	↓	↓			↓	↓	↓
FABP4	↓		↓								↓	↓	↓			
IL 18	↓	↓		↓		↓	↓			↓		↓			↓	↓
LPL	↓	↓	↓					↓				↓	↓			
MMP9		↓	↓		↓	↓	↓	↓	↓	↓	↓			↓	↓	↓
PLAT	↑	↑													↑	

* *Abbreviations for upstream regulators: RGS2, regulator of G protein signaling; PTGS2, Prostaglandin-endoperoxide synthase 2; PRTN3, Proteinase 3; HRG, Histidine Rich Glycoprotein; ERK1/2, extracellular signal–regulated kinases 1/2; GH, Growth Hormone; IFNαR, Interferon α Receptor; TLR4, Toll-like receptor 4*.

† *Abbreviations for genes: CCL2, C-C motif ligand 2; FABP4, fatty acid binding protein 4; LPL, lipoprotein lipase; MMP9, Matrix metallopeptidase 9; PLAT, Plasminogen Activator. The ↑ arrow is for genes that were up-regulated and the ↓ arrow is for a gene that was down-regulated*.

**Table 5:** Upstream regulators in mesenteric adipose tissue and how they influence gene expression.

**Genes^†^**	Upstream Regulators^*^														
	**Top 5**					**IL1A**	**IL2**	**IL4**	**IL6**	**IL18**	**IGF-1**	**GH**	**TLR 4**	**PRL**	**ERK 1/2**
	**TNF**	**IFNAR**	**IL-1B**	**NF-κβ**	**IFNγ**										
B2M	↓	↓	↓		↓										↓
CCL11	↑		↑		↑		↑	↑	↑	↑	↑				
CCND1	↓			↓			↓		↓		↓	↓		↓	↓
COL1α1			↑		↑						↑	↑		↑	
CXCL2	↓		↓	↓	↓	↓			↓				↓		
GADD45	↑						↑	↑	↑		↑		↑		
GBP2	↓	↓		↓	↓			↓	↓				↓		
IDO1	↓	↓		↓	↓			↓	↓	↓					
IL1A	↓		↓	↓	↓	↓	↓			↓			↓		↓
IRF1	↓	↓	↓	↓	↓		↓	↓	↓			↓	↓	↓	
JUNB	↓			↓	↓				↓			↓			↓
MMP1	↓		↓	↓	↓	↓		↓		↓			↓		↓
MYC	↑			↑	↑		↑	↑	↑		↑	↑		↑	↑
NFκβIα	↑		↑	↑	↑	↑		↑	↑			↑	↑		
PSMB8	↓	↓			↓				↓		↓				↓
PSMB9	↓	↓		↓	↓				↓		↓				↓
RSAD2		↓		↓	↓								↓	↓	
TAP1	↓	↓		↓	↓				↓		↓				↓

* *Abbreviations for upstream regulators: IFNαR, interferon α receptor; GH, growth hormone; TLR 4, toll-like receptor 4; PRL, prolactin; ERK1/2, extracellular signal–regulated kinases 1/2*.

† *Abbreviations for genes: B2M, beta-2-microglobulin; CCL11, C-C motif ligand 11; CCND1, cyclin D1; COL1α1, collagen type 1 α chain 1; C-X-C motif, chemokine; CXCL2, ligand 2; GADD45, growth arrest and DNA damage 45; GBP2, guanine binding protein 2; IDO1, indoleamine 2,3-dioxygenase 1; IRF1, interferon regulatory factor 1; MMP1, Matrix metallopeptidase 1; NFκβIα, NF-κβ inhibitor α; PSMB8, proteasome subunit beta 8; PSMB9, proteasome subunit beta 9; RSAD2, radical S-adenosyl methionine domain containing 2; TAP1, transporter 1. The ↑ arrow is for genes that were up-regulated and the ↓ arrow is for a gene that was down-regulated*.

Functional analysis of all significant (*P* < 0.05) genes, with and without 1.5 fold change within the datasets (BROMO and CON) was done via IPA library. Functions related to cell movement, cell development, cell growth and proliferation, cell death, and overall cellular function and maintenance were the top 5 functional molecular categories (*P* < 0.001) affected by BROMO treatment within the intestinal epithelium (Table 6). Whereas, hematological system development and function, immune cell trafficking, tissue morphology, skeletal and muscular system development and overall tissue development were the top physiological function categories within the epithelium of the intestine depressed (*P* < 0.001) in BROMO steers (Table 6). In the intestinal epithelium all individual functions were depressed (*P* < 0.001) in BROMO steers compared with CON steers (Table 7). Most of these functions were related to immune cell activation and immune response such as: cell movement of neutrophils, infiltration, activation, and accumulation of phagocytes, activation and accumulation of myeloid cells, quantity of macrophages, and overall inflammation. Other functions were related to metabolism, quantity of carbohydrates and proteins, synthesis of lipids, organismal survival, development of epithelial tissue, and vasculogenesis (Table 7).

**Table 6:** IPA^*^ bio-functions enriched by significantly expressed genes in the intestinal epithelium of bromocriptine treated Holstein steers.

**Top 5 molecular and cellular functions**	**Top 5 physiological system development and functions**
Cellular Movement	Hematological System Development and Function
Cellular Development	Immune Cell Trafficking
Cellular Growth and Proliferation	Tissue Morphology
Cell Death and Survival	Skeletal and Muscular System Development and Function
Cellular Function and Maintenance	Tissue Development

* *IPA, Ingenuity Pathway Analysis*.

**Table 7:** Individual functions^*^ activated or depressed within the intestinal epithelium of Holstein steers treated with bromocriptine compared to saline treated steers.

**IPA^†^ Bio-function**	**Functional effect**	***P*-value**	**z-Score of activation^‡^**	**Genes, *n***
Carbohydrate Metabolism	Quantity of carbohydrate	0.0017	−1.176	4
Cardiovascular System Development	Vasculogenesis	0.0009	−1.073	5
Cell-To-Cell Signaling	Activation of cells	0.0015	−1.556	5
	Activation of myeloid cells	<0.0001	−1.282	4
	Activation of phagocytes	0.0001	−1.282	4
Cellular Movement	Infiltration by myeloid cells	<0.0001	−1.445	5
	Chemotaxis	0.0018	−1.133	4
	Cell movement of neutrophils	<0.0001	−1.695	5
	Cellular infiltration by phagocytes	<0.0001	−1.067	5
	Migration of smooth muscle cells	<0.0001	−1.282	4
Immune Cell Trafficking and Inflammatory Response	Accumulation of phagocytes	<0.0001	−1.067	4
	Quantity of macrophages	<0.0001	−1.612	4
	Accumulation of myeloid cells	<0.0001	−1.192	4
	Inflammatory response	0.0008	−1.67	5
	Inflammation of absolute anatomical region	<0.0001	−1.533	7
	Inflammation of organ	<0.0001	−1.414	10
	Synthesis of lipid	0.0043	−1.963	4
Organismal Survival	Survival of organism	0.0009	1.138	5
Protein Synthesis	Metabolism of protein	0.0026	−1.927	5
	Quantity of protein in blood	0.0018	−1.06	4
Tissue Development	Development of epithelial tissue	0.0001	−1.103	5

* *Functions were selected upon the criteria of having a P value < 0.05 and an absolute value for the z score of activation of > 1*.

† *PA, Ingenuity Pathway Analysis*.

‡ *A negative z score of activation indicates the function was depressed and a positive z score of activation indicates the functions was activated*.

Ingenuity pathway analysis of mesenteric adipose determined that the top molecular categories were antigen presentation, protein synthesis, cell death, cell movement, and cell to cell signaling and interaction (Table 8). Whereas, the top physiological function categories were organismal survival, hematological system development and function, tissue morphology, organismal development, and immune cell trafficking (Table 8). The complete list of individual functions influenced by BROMO treatment in the mesenteric adipose is provided in Supplementary Table 1. Physiologically relevant functions to the hypothesis of the study (*P* < 0.001) related to metabolism, tissue and cell health and growth, cell movement, and some immune and inflammatory responses were down regulated (*P* < 0.001; Table 9). The few immune response functions that showed activation (*P* < 0.001) with BROMO treatment were recruitment of macrophages, activation of myeloid cells and leukocytes, and proliferation of mononuclear leukocytes and lymphatic system cells. There were multiple gastrointestinal disease functions, focused largely around inflammation, activated due to exposure to BROMO including enteritis and colitis (Table 9). Functions that were related to metabolism consisted of mainly lipid metabolism functions, transport of lipid, cholesterol, and steroids and the quantity of HDL in the blood. Cellular movement and migration of both epithelial and smooth muscles cells was depressed in BROMO steers compared with CON steers (Table 9).

**Table 8:** IPA^*^ bio-functions enriched by significantly expressed genes in mesenteric adipose of bromocriptine treated Holstein steers.

**Top 5 molecular and cellular functions**	**Top 5 physiological system development and functions**
Antigen Presentation	Organismal Survival
Protein Synthesis	Hematological System Development and Function
Cell Death and Survival	Tissue Morphology
Cellular Movement	Organismal Development
Cell to Cell Signaling and Interaction	Immune Cell Trafficking

* *IPA, Ingenuity Pathway Analysis*.

**Table 9:** Individual functions^*^ activated or depressed within mesenteric adipose of Holstein steers treated with bromocriptine compared to saline treated Holstein steers.

**IPA^†^**	**Functional effect**	***P*-value**	**z-Score of activation^‡^**	**Genes, *n***
**Bio-function**				
Antigen Presentation	Activation of leukocytes	<0.0001	1.028	19
	Proliferation of mononuclear leukocytes	0.0041	2.012	18
	Proliferation of lymphatic system cells	0.0040	1.8	19
Gastrointestinal Disease	Gastroenteritis	0.0014	1.588	13
	Enteritis	0.0025	1.352	12
	Colitis	0.0031	1.741	11
	Inflammation of gastrointestinal tract	0.0016	1.588	13
	Abnormality of large intestine	0.0024	1.969	12
Hematological Disease, Development, and Function	Blood protein disorder	0.0008	1.954	16
	Lymphoproliferative disorder	<0.0001	1.475	47
	Quantity of hematopoietic progenitor cells	0.0012	−1.226	14
	Quantity of phagocytes	<0.0001	−1.35	16
	Quantity of macrophages	<0.0001	−1.117	11
Hematological System Development and Function	Quantity of T lymphocytes	0.0035	−1.476	15
	Quantity of lymphocytes	0.0011	−1.084	20
	Quantity of blood cells	<0.0001	−2.148	34
	Quantity of granulocytes	0.0004	−1.551	12
	Quantity of leukocytes	<0.0001	−1.509	29
	Quantity of mononuclear leukocytes	0.0007	−1.399	21
	Quantity of myeloid cells	<0.0001	−1.144	18
Humoral Immune Response	Quantity of immunoglobulin	0.0022	−1.261	10
Inflammatory Disease and Response	Inflammation of body cavity	<0.0001	1.258	26
	Inflammation of absolute anatomical region	<0.0001	1.97	33
Organismal Development	Growth of organism	0.0006	1.16	22
Organismal Injury and Abnormalities	Cytosis	0.0011	−1.362	12
Tissue Morphology	Quantity of cells	<0.0001	−1.519	49

* *Functions were selected upon the criteria of having a P value < 0.05, more than 10 genes identified within, and an absolute value for the z score of activation of > 1*.

† *IPA, Ingenuity Pathway Analysis*.

‡ *A negative z score of activation indicates the function was depressed and a positive z score of activation indicates the functions was activated*.

## Discussion

The widespread usage of tall fescue for grazing animals makes the decrease in productivity due to fescue toxicosis economically important to beef production. Tall fescue (*Lolium arundinaceum*) has a symbiotic relationship to a fungal endophyte (*Epichloë coenophiala*) which infects mainly the seed head of the plant but also confers growth and heartiness advantages which increase the usefulness of tall fescue as a grazing feed source (1). The endophytic infection creates a source of ergot alkaloids of which ergovaline is found in the greatest concentrations (26). Consumption of these associated ergot alkaloids in sufficient amounts to cause fescue toxicosis in cattle results in a dramatic reduction in circulating prolactin concentration; a diagnostic tool used to confirm exposure to detrimental ergot alkaloids (5, 12–15, 20). Bromocriptine, a synthetic ergopeptine and dopamine receptor agonist, shares a high degree of structural homology with ergovaline and has also been shown to reduce circulating prolactin concentration in cattle (14, 20). More recently we have shown that 90% of DEG (*n* = 866) in mammary tissue respond similarly for both fed fescue-derived alkaloids and BROMO treated cows (15); indicating that BROMO provides an adequate model for studying physiological changes in cattle grazing endophyte-infected tall fescue. In the current study we extend our finding concerning the effects of BROMO on the transcriptome to the intestinal epithelium and mesenteric adipose in growing ruminant animals.

### Intestinal Epithelium

Based on the current dataset and previous work (15, 20) in dairy cows BROMO impacted the intestinal transcriptome less than that observed in the transcriptome of mesenteric adipose and the mammary gland. We cannot eliminate the possibility that the intestinal transcriptome could be differentially influenced by luminal vs. systemic administration of ergot alkaloids, however, previous studies have shown that subcutaneous injections of bromocriptine alter intestinal transit in mice (27) and both intravenous and intraluminal bromocriptine stimulated small intestinal absorption of nutrients in multiple mammalian species (28). Based on selection criteria, RNA-Seq analysis revealed two DEG within the intestinal epithelium. These genes were fatty acid binding protein 4 (FABP4) which was down regulated and UDP-GlcNAc:betaGal Beta-1,3-N-Acetylglucosaminyltransferase 6 (B3GNT6) which was up regulated in steers treated with BROMO. Fatty acid binding protein is known to be involved in transport and metabolism of long chain fatty acids and overall lipid metabolism (11, 29, 30). Up regulation of B3GNT6 is indicative of increased lymphocyte trafficking and homing which may suggested increased immune activity as reported in other published data (31). Interestingly, although a limited number of DEG were detected both fit with previously reported effects of ergot alkaloid exposure.

There were two canonical pathways (LXR/RXR activation, granulocyte adhesion and diapedesis pathways) depressed in both tissue types. These pathways provide further evidence that alkaloid exposure negatively impacts immune function. Granulocyte (neutrophils, basophils, and eosinophils) adhesion and diapedesis are more involved in the efficiency and activity of the immune response within these tissues. This is supported by depressed movement of neutrophil identified by individual function analysis within IPA. Most of the other intestine specific pathways influenced by alkaloid exposure have a role in the inflammatory response: the inflammasome pathway, HMGB1, and atherosclerosis signaling. Interestingly, HMGB1 signaling and monocyte infiltration can also influence expression of adhesion molecules (32). While this was not evident in DEGs, upon further analysis we found both IL18 and C-C Motif Chemokine Ligand 2 (CCL2) tended to be down regulated and plasminogen activator (PLAT) tended to be up regulated. These genes were sufficient to identify the HMGB1 pathway as significantly affected and may suggest that BROMO treatment may impact barrier function of the intestine similar to results in mice (33).

Cellular and physiological functions identified from these datasets were indicative of effects on cell movement, cell and tissue growth and development, and cell survival. Functional analysis using IPA indicated migration of smooth muscle cells was influenced in BROMO steers. This is meaningful in that intravenous administration of ergot alkaloids has also resulted in an immediate inhibition of muscle contractions in the forestomach of ruminants (34). Thus, these effects could be due to direct actions of alkaloids on myenteric neurons and on smooth muscle cells (35). The most significant physiological function identified was hematological system development and function. Initially this was thought to be changes in vascularity which would agree with previously published research (2, 10, 36–39). However, a closer look at individual functions revealed that circulating immune surveillance rather than angiogenesis was impacted within the intestinal epithelium. Because vasoactivity was not measured in the current study it is equivocal whether fescue alkaloid-induced vasoconstriction, i.e., predominantly ergovaline, fails to result in DEG or whether the dose of BROMO used in the current study was below the threshold to induce vasoconstriction in visceral blood vessels as previously reported. The latter can be supported by the fact that the dose of BROMO used in the current study had no impact on the ability of treated steers to consume 1.5 × net energy for maintenance daily. Whereas, previous studies using fescue seed have shown much more severe effects on feed intake in cattle (8, 9, 11).

### Mesenteric Adipose

There was a greater number of DEG due to BROMO in the mesenteric adipose with 20 DEG (14 down regulated and 6 up regulated). Down regulated genes functions included enzymes, transporters, ion channel, cytokines, and other immune response molecules. The 4 DEG identified as an enzymes were Cytochrome P450 Family 4 Subfamily F Member 2 (CYP4F2), involved in fatty acid and cholesterol synthesis; Activation Induced Cytidine Deaminase (AICDA), regulation of immunoglobulin somatic hypermutation and class switch recombination (CSR), processes required for B-cell development of high affinity antibodies; cadherin 16 (CDH16) an adhesion molecule, and Glycosyltransferase 1 Domain Containing 1 (GLT1D1) for which an exact function is unknown. The transporters down regulated in adipose were both members of the solute carrier family, SLC9A2 and SLC26A5, and are involved in cation and anion transport, respectively. In addition to SLC9A2, which is a sodium/hydrogen exchanger, Acid Sensing Ion Channel Subunit Family Member 5 (ASIC5) a Na^+^ ion channel was also down regulated. The down regulation of SLC9A2 which functions to counter adverse environmental conditions, specifically pH regulation is intriguing but the exact role cannot be determined in the current dataset.

Maruo et al. (40) suggested activation of a negative feedback mechanism in the intestine and liver of pigs fed ergot alkaloids which also had a down regulation of toll-like receptors and a variety for cytokines including IL-6 and Tumor Necrosis Factor α (TNFα). In pigs fed ergot alkaloids tissues may be attempting to restore barrier function by up regulating mRNA expression of adhesion proteins such as occludins, claudins 3 and 4, and others (40). Accordingly, the down regulation of most genes in the current study may exemplify this purposed feedback mechanism within the adipose and reflect the drive to return to normal physiological conditions. In contrast, alkaloid ingestion has been reported to cause increased inflammation (31) and decrease overall immune competency (41). These conflicting results were also apparent in our data. However, an explanation is not immediately apparent but there are still clear alterations in immunological response and function.

The six up regulated DEG had identified functions of enzyme activity [Monooxygenase DBH Like 1 (MOXD1)], molecular transport [Sortilin Related VPS10 Domain Containing Receptor 2 (SORCS2); Solute Carrier Family 11 Member 1 (SLC11A1)], transmembrane receptor [Delta/Notch Like EGF Repeat (DNER)], cytokine [Platelet Factor 4 (PF4)] and other unknown functions [Lymphocyte Antigen 6 Family Member G5B (LY6G5B)]. In contrast, mRNA and protein expression in the intestine and liver of swine fed ergot alkaloids exhibited up regulation of adhesion molecules (40); however, CSH16 was the only DEG identified that has adhesion properties and it was down regulated. This may be a differential effect of alkaloid exposure between species but may simply confirm the wide variety of physiological functions influenced by ergot alkaloids.

The canonical pathways influenced by BROMO treatment were largely focused on inflammation, immune response, and lipid metabolism. Pathway identification from IPA utilized all significantly expressed genes (*P* < 0.05) with and without a fold change >1.5. The LXR/RXR activation and granulocyte adhesion and diapedesis pathways were depressed in the mesenteric adipose as well as the intestine. Inhibition of the LXR/RXR heterodimer would directly modulate the initiation of immune and inflammatory responses in macrophages (42). Individual functions support the influence on macrophage function from recruitment and movement to proliferation and overall quantity. Whereas, granulocyte (neutrophils, basophils, and eosinophils) adhesion and diapedesis are more involved in the efficiency and activity of the immune response within these tissues. Unlike in the intestine, overall granulocyte quantity and recruitment were depressed in adipose tissue of these steers rather than neutrophils specifically. Genes involved with the compliment system (SERPINB2 and C8A), chemokine secretion (CXCL2 and PF4), and immunoglobulin function (AICDA and HEPACAM2) were all down regulated DEG except for PF4 which was one of a fed up regulated DEG in adipose tissue. Further work needs to be completed to fully understand the role and mechanisms of DEG within these pathways and how ergot alkaloid exposure changes the function of these pathways and overall physiology of the animal.

Accumulation of ergot alkaloids in adipose tissue has been reported to disrupt or alter lipid metabolism (30). Both CYP4F2 and NR1H4 were down regulated in the current dataset. Lipid and bile acid synthesis were depressed with down regulation of CYP4F2 and NR1H4 and further supported by identification of HDL and LDL inhibition within LXR/RXR pathways. These data begin to identify specific genes and a putative mechanism to explain how alkaloids, specifically BROMO, influence adipocytes and lipid metabolism.

LPS/IL-1 mediated inhibition of RXR and IL-6 signaling were activated and FXR/RXR activation, antigen presentation, and dendritic cell maturation were depressed by alkaloid exposure. Canonical pathways influenced by BROMO treatment, like DEG, indicate opposing effects on immune response. Specifically, the influence of ergot alkaloids on immune cell trafficking, cellular movement, and immune response within abdominal tissues. Previously reported data demonstrated that ergot alkaloids increased inflammation but decreased overall immune function in steers that grazed endophyte-infected tall fescue as evidenced by lower Cu status, depressed immune-competency, decreased major histocompatibility complex class II expression, and macrophage phagocytosis activity (41). The current dataset agrees with previous literature (41) that exposed cattle could have a decreased immune response through inhibition of associated genes and other pathways associated with a variety of mechanisms. However, our data doesn't supply any further evidence for increased inflammation (31, 41). This dichotomy in the literature is similar to the conflicting DEG, pathways, and functions observed in the current dataset and thus requires more work to completely elucidate the mechanisms behind this contradiction.

In the current dataset, the serotonin degradation pathway (Table 2) was depressed by BROMO treatment in the adipose which is consistent with the known interaction between bromocriptine (43) or alkaloids (44) and serotonin metabolism. Specifically, ergot alkaloids have been reported to interact with serotonin receptors in vascular smooth muscle (19, 45, 46). Expression of the serotonin receptor 5HTR2A and 5HTR4 in gastrointestinal smooth muscle was reduced in steers treated with ergot alkaloids compared with control steers (47). Serotonin receptor down regulation is likely due to alkaloids binding to the receptor. Bromocriptine has a strong affinity for the 5HTR2A receptor (48). Therefore, BROMO is likely inhibiting the serotonin degradation pathway through antagonistic activity within the mesenteric adipose after exposure to BROMO. These data and that from Klotz et al. (47) suggest an interactive role of the adipose, intestine, and ergot alkaloids throughout the serotonin pathway from ligand to receptor.

Surprisingly, there were limited pathways identified by the current dataset specifically involved with angiogenesis or vascularity. As discussed earlier, alkaloid exposure has been reported to constrict blood vessels (2) and flow (10, 36–39) in other datasets. None of the DEGs identified had a direct effect on vascularity but CD8A, CXCL2, C8A, SERPINB2, AICDA, HEPCAM2, and PF4 are directly involved in immune system response and function. This may suggest that abdominal effects, such as necrotic fat, are a result of persistent activation of the immune system vs. vasoconstriction as seen in peripheral tissues (2, 6, 7, 9).

In the mesenteric adipose compared with the intestinal epithelium there was a greater number and variety of functions influenced by BROMO treatment; however most functions identified represented cell movement and survival, immune cell trafficking, tissue/organismal development, morphology, and survival. Mulac and Humpf (49) demonstrated apoptotic effects of ergot alkaloids on human primary cells in culture which may help to explain the inhibition of cell survival. Functions related to overall quantity, recruitment, and movement of macrophages along with other leukocytes were identified by functional analysis in the current dataset. Macrophages within adipose tissue are generally found scattered or in crown-like structures around dead adipocytes (48). These crown-like structures were differentially expressed in abdominal fat depots compared with subcutaneous and pelvic adipose of obese (50) or leptin and leptin receptor deficient mice (51). These structures within adipose may be a mechanism responsible for necrotic fat depot development but more likely are functioning to clear necrotic adipocytes. In the current dataset, more work needs to be completed to know if the macrophages were in crown-like structures associated with deceased adipocytes and if this a possible mechanism in the formation of necrotic fat depots rather than vasoconstriction.

The influence of BROMO on lipid metabolism is likely due, indirectly, to the decrease in prolactin (52). Connor et al. (53) found exposure to shortened day lengths decreased circulating prolactin which led to decreased acyl-CoA dehydrogenase. As the first step in β-oxidation acyl-CoA dehydrogenase would reduce fatty acids (53) but have also been associated with increased hepatic triacylglycerol (54) and non-esterified fatty acids (54, 55). Alterations in lipid metabolism due to bromoergocriptine did not impact milk fat in lactating dairy cows (56). Thus, indicating prolactin alters fatty acid (52) and cholesterol (8, 57) metabolism. However, in addition to those mediated through prolactin, it is possible that BROMO treatment and ergot alkaloids have a more direct effect on lipid metabolism. These data also indicate BROMO can alter adipose metabolism and function which would change the morphology of adipose tissue and influence immune surveillance by leukocytes located within this tissue.

In summary, exposure of Holstein steers to systemic bromocriptine resulted in decreased plasma prolactin concentration which is what occurs when steers graze endophyte-infected tall fescue or are administered ergot alkaloids. RNA-Seq analysis revealed that exposure to a synthetic alkaloid was influential on pathways and functions involved with immune cell trafficking, inflammatory responses, and lipid metabolism compared with other physiological pathways and functions. These data provide insight into the specific tissue responses and potential pathways and genes affected and thereby begin the process of elucidating the mechanisms behind compromised health and productivity of animals grazing ergot alkaloids. However, further and more specific analyses need to be conducted to completely understand the mechanisms behind the negative effects of animals exposed to ergot alkaloids.

## Data Availability Statement

The raw data reads were lost and are not available for upload, however the extended and comprehensive findings have been provided in the supplemental tables. Any further information that we can provide will be available upon discussion with the authors.

## Ethics Statement

The animal study was reviewed and approved by University of Kentucky Animal Care and Use Committee.

## Author Contributions

KMcLea and KMcLeo performed experiment and conception and design of research. KMcLea, RB, CL, JE, and KMcLeo analyzed data. KMcLea, RB, CL, and KMcLeo interpreted results of experiments. KMcLea prepared figures and drafted manuscript. KMcLea, RB, CL, JK, and KMcLeo edited and revised manuscript. KMcLea, RB, CL, JK, JE, and KMcLeo approved final version of manuscript. All authors contributed to the article and approved the submitted version.

## Conflict of Interest

The authors declare that the research was conducted in the absence of any commercial or financial relationships that could be construed as a potential conflict of interest.
